# Hamid Jafari: the polio endgame and its challenges

**DOI:** 10.2471/BLT.24.031124

**Published:** 2024-11-01

## Abstract

Hamid Jafari talks to Gary Humphreys about the non-linear nature of progress toward polio eradication and the challenges faced in the last two polio-endemic countries in the world.

*Q: The International Health Regulations (IHR)(2005) Emergency Committee for Polio recently expressed concern about the resurgence and spread of wild poliovirus (WPV1) in Afghanistan and Pakistan. What can be done to reverse those trends?*

A: First, regarding the question of trends. Progress towards eradication tends not to be linear because of the formidable challenges faced in vaccinating all children in certain contexts and the nature of poliovirus which, when not interrupted, causes periodic outbreaks. The trend in reported cases in Pakistan is a perfect example of this. There was a steep drop from 306 reported cases in 2014 down to 8 cases in 2017, a bounce back to 147 cases in 2019 and just one case in 2021. This year we already have 28 cases and expect to see more. However, these cases in no way predict what is going to happen in the next 12–15 months. As Nelson Mandela once said, “It always seems impossible until it is done,” an epithet that I consider to be particularly applicable to polio eradication – a view I derive from lived experience. In India we also had to deal with an end game that seemingly would not end. By the year 2000, every state had stopped endemic transmission with the exceptions of Uttar Pradesh and Bihar. It took another 11 years, with periodic outbreaks, to stop transmission in those two states concurrently, and just as in Afghanistan and Pakistan, the biggest challenge was reaching all the children.

*Q: What proportion of children need to be vaccinated to achieve herd immunity?*

A: This depends on the local epidemiological conditions for poliovirus transmission. For example, in sparsely settled areas, better conditions of hygiene and in a temperate climate, 80% to 90% vaccination coverage rates may be sufficient. Achieving herd immunity to stop transmission in places like Uttar Pradesh and Bihar and historic reservoirs of Pakistan or northern Nigeria require coverage rates that must be sustained well above 95%. You can make all the progress you want on water, sanitation and hygiene but if you are not vaccinating more than 95% children consistently you will not put a stop to transmission. We managed to do that in India despite obstacles that included very poor sanitation conditions in the Gangetic plain, intestinal infections that interfered with the uptake of the oral vaccine and extremely high birth rates. In Uttar Pradesh alone, the birth cohort was half a million children every month and we were in a race to get the vaccine to children before the virus got to them. There were many public health experts who said that it was technically and biologically not feasible to eradicate polio in India – a view that seemed credible during periodic outbreaks of polio, including the one in 2009 when 741 cases were reported. Just over a year later, polio was gone. The last case was reported in January 2011, and it has not come back.

*Q: But aren’t the challenges faced in Afghanistan and Pakistan different, notably in terms of tribal conflict and militancy?*

A: There are in fact a mix of challenges, ranging from epidemiological conditions that favour poliovirus transmission to community resistance, bargaining boycotts (where stakeholders halt programmes as a bargaining strategy) and militancy. In Pakistan, some areas along the 2600-kilometre border with Afghanistan are particularly hard to access due to security concerns and social upheaval. These factors, along with extensive and often unpredictable population movements, have resulted in many children being missed during vaccination campaigns. There have also been issues related to the pressure to achieve eradication, which has given rise to tensions in some communities and fear among workers, resulting in a lack of data transparency. There have also been instances of under-reporting of the number of missed children in certain pockets of historic reservoirs in Pakistan. 

*Q: What are you doing to address these different challenges?*

A: We are taking a multifaceted approach, starting with ensuring strong government commitment to mitigate contextual challenges, including the provision of enhanced security measures for vaccination teams, and engaging with local communities. The use of female vaccination workers has been essential for success. Recruiting women has always been a priority because women can access households more effectively. However, this has not always been simple. For example, in 2019, I went to Kandahar, a city in the south of Afghanistan, and found that only one in five of the health workers were women. When we asked the Emergency Operations Centre about this, we were told that female participation in the workforce was discouraged for cultural reasons and that families didn’t allow their daughters to go out unaccompanied. However, one of my female colleagues got into a conversation with some young Afghan women sitting in a separate room at the centre, and they said they were college graduates and keen to work, and that it wasn’t their families who were blocking them but the men in the vaccination programme. They said their families would allow them to work, especially if their supervisor was a female. So, within a year, we went from 20% frontline workers being females to 80%. With no house-to-house campaigns, unfortunately female vaccinators are no longer engaged in the south.

The programme in Pakistan is in a ‘reset’ phase that includes improving programme coordination and management within and across the national and provincial emergency operations centres. Efforts are also being made to redefine and remap migrant and mobile populations that now include a wider range of mobile groups, including migrants from border areas and labour moving to and from historic polio reservoirs and different nomadic groups. Local cross-border coordination is being optimized, and vaccination at major border crossing points is being rigorously implemented on both sides. Regarding data transparency and accuracy, we are intensifying objective monitoring and rotating workers and their supervisors. More broadly speaking, we are addressing the various socio-political and security challenges that hinder consistent immunization coverage. And we are getting better at it. The programme is not what it was 10 years ago or even five years ago. It is adapting to the evolving political, social and security challenges.

*Q: Can you give a couple of ways in which the programme is adapting?*

A: There have been many adaptations around security and access, building bridges with communities, reaching the hard-to-reach mobile populations and building confidence among frontline workers. We've placed considerable emphasis on building trust as a crucial component of our vaccine distribution efforts, partly by ensuring that vaccination teams communicate in the appropriate languages, thereby preventing any misunderstandings on the doorstep. This has been particularly important in Karachi, home to significant Pashtun migrant communities from Pakistan and Afghanistan. We have also worked to better engage with local players. In Pakistan, for example, we engage with the army and civilian leaders to optimize coordination and enhance security. Greater regional engagement, solidarity and collaboration has also come about with the establishment of a ministerial Regional Subcommittee for Polio Eradication and Outbreaks which was set up through a Regional Committee resolution in 2020. The subcommittee has not only encouraged regional solidarity, it has allowed for the establishment of backchannels for resolving difficult political and access challenges, such as when a transactional approach by one minister halted a vaccination campaign and a senior minister in the region stepped in to resolve the impasse.

*Q: The IHR Emergency Committee for Polio also drew attention to vaccine-derived polio virus outbreaks which are on the increase in several countries, notably in Africa. Have these been an issue in Afghanistan and Pakistan?*

A: There are currently active outbreaks of cVDPV2 (circulating vaccine-derived poliovirus type 2) in Somalia, Sudan, Yemen and most recently in Gaza, where I am pleased to say high levels of coverage were achieved. Pakistan had an outbreak of cVDPV2 in Baluchistan in 2017, the year after the withdrawal of the trivalent oral polio vaccine, which was controlled very rapidly. A more extensive outbreak occurred in 2019–2020, which started in northern Pakistan and then spread to Afghanistan, but both countries responded aggressively and stopped that outbreak despite the COVID-19 pandemic. The experience in the Eastern Mediterranean region has been relatively positive, the one exception being Somalia, where the longest running cVDPV2 outbreak continues. There has been a decline in quality of campaigns there – now being addressed – and challenges reaching communities in areas under the control of militants.

*Q: There has been much criticism of the bivalent oral polio vaccine as a driver of vaccine-derived polio outbreaks. Is enough being done to phase in the novel oral polio vaccine (nOPV2), its replacement?*

A: WHO approved nOPV2 under Emergency Use Listing in November 2020 and it was pre-qualified by WHO in December 2023. However, it would be a mistake to consider nOPV2 a panacea. The vaccine virus may be genetically more stable and so associated with a much lower risk of new cVDPV2 emergence, but here again the key is reaching enough children to achieve high coverage that stops outbreaks and precludes the emergence of cVDPV2 infections. We have repeatedly learned that polio persists and spreads in situations where we see ‘failure to vaccinate’, rather than ‘vaccine failure’ which is vanishingly rare.

*Q: To what extent do the reductions in funding from key donors such as the United Kingdom of Great Britain and Northern Ireland pose a risk to maintaining the gains achieved in polio eradication efforts?*

A: The donors to the global polio eradication effort have been exceptional, maintaining funding against the backdrop of significant budget reductions for development and global health. While the United Kingdom government did drastically reduce its funding, it has increased its political advocacy for eradication in Parliament and within the G7 and has secured basic funding, even as it awaits approval for a large contribution in line with its historic support. Major new regional donors have also come forward, notable among them Saudi Arabia, and we fully expect the United Arab Emirates to renew their funding commitment. Rotary International’s leadership remains deeply committed, but they face the challenge of keeping Rotarians worldwide engaged in the face of competing humanitarian priorities. For the time being, Rotary has sustained its funding and advocacy efforts at high levels. Their contributions remain invaluable, and I hope they understand how critical their support is in addressing the complex ‘last mile’ problems and challenges we face. The task may seem impossible now, but it will cease to appear so, indeed be so, the day it is accomplished.

